# DESA-YOLO: A Growth-Stage Adaptive Pig Face Recognition Algorithm Based on Multi-Scale Feature Fusion

**DOI:** 10.3390/ani16101468

**Published:** 2026-05-10

**Authors:** Xin Li, Jinghan Cai, Tonghai Liu, Fanzhen Wang, Xiaomeng Zheng, Meng Wang

**Affiliations:** 1 College of Computer and Information Engineering, Tianjin Agricultural University, Tianjin 300392, China; 18238743259@163.com (X.L.); q1463747994@163.com (J.C.); 15735086688@163.com (X.Z.); 18336692532@163.com (M.W.); 2 College of Engineering Technology, Tianjin Agricultural University, Tianjin 300392, China

**Keywords:** YOLO11, individual recognition, pig face recognition, growth stages

## Abstract

This paper proposes a pig face individual recognition algorithm named DESA-YOLO based on an improved YOLO11 model, aiming to address the adaptability issue of pig face recognition across different growth stages. With the large-scale development of pig farming, traditional individual identification methods suffer from low efficiency and high cost, while pig face recognition technology has great application potential as an important tool for precision suckling and disease prevention. Due to the significant facial feature differences among pigs at different growth stages, this study proposes an improved YOLO11 architecture to address this challenge. The method improves detection accuracy and adaptability by introducing a DualConv structure, an EMA module, a SEAM attention mechanism, and an ASFF detection head. Experimental results show that DESA-YOLO achieves significant improvements over traditional models such as YOLOv5 and YOLOv8 in precision, recall, mAP, and F1 score, obtaining an mAP of 93.7%, which represents increases of 6.3%, 3.5%, and 3% in precision, recall, and mAP respectively compared with the YOLO11 baseline model. Ablation experiments and heatmap visualizations further validate the effectiveness of the proposed improvement modules. The improved model demonstrates higher adaptability and stability across different pig growth stages, while maintaining real-time inference performance for practical deployment.

## Simple Summary

This study addresses the challenge of accurately recognizing. under growth-stage-related appearance variations, which is essential for efficient and large-scale pig farming. Traditional identification methods are costly and inefficient, while existing recognition approaches struggle with the significant facial changes that occur as pigs grow. To solve this problem, this study proposes a new method called DESA-YOLO, designed to improve recognition accuracy and adaptability under representative appearance variations from suckling to fattening stages. Experimental results show that the proposed method achieves higher accuracy, precision, and recall than existing models, especially during the weaning stage where recognition is most difficult. The results demonstrate that the method can reliably identify individual pigs under varying conditions. This research provides a practical solution for improving animal monitoring, suckling management, and disease prevention. It can help farmers reduce labor costs, increase efficiency, and support the development of intelligent farming systems, contributing to more sustainable and modern livestock production.

## 1. Introduction

In modern swine production, precise individual identification is crucial for achieving scientific husbandry management, disease prevention and control, and production performance evaluation. However, traditional methods—such as manual observation and radio frequency identification (RFID) technology [1]—suffer from inefficiencies, high costs, error-prone operations, and stress-inducing effects on pigs, making them inadequate for meeting the automation and intelligent management demands of large-scale farms. These limitations make them ill-suited for large-scale operations [2]. Among these, pig face recognition technology has garnered significant attention as a key method for achieving precise individual identification. In recent years, with the advancement of deep learning techniques in computer vision [3,4,5], numerous studies have focused on developing efficient pig face recognition systems to meet practical application needs in complex farming environments. This technology helps reduce property losses [6]. Pig face recognition has emerged as a core method for precision livestock management, disease monitoring [7], and growth status tracking, has gradually become a key research direction for the intelligent transformation of the livestock industry. The core of pig face recognition technology lies in accurately detecting the pig face region from images and further identifying individual identities [8]. However, facial features undergo significant changes across different growth stages—piglets exhibit rounder faces, while adults display more defined contours and wrinkles—posing substantial challenges for recognition. Furthermore, practical image acquisition is subject to variations in lighting conditions, shooting angles, and background interference, further complicating identification accuracy.

Wang and Liu [9] proposed a two-stage pig face recognition method based on deep convolutional neural networks. This approach achieves a classification accuracy of 96.8% by detecting pig faces using the EfficientDet-D0 model and combining it with a triplet margin loss function for classification. However, the method incurs high computational costs and demands stringent hardware resources, making deployment in resource-constrained environments challenging. Li et al. [10] designed a lightweight cattle face recognition model that significantly reduces model parameters and computational complexity through global average pooling (GAP) and stride-2 convolutions. While achieving 98.37% accuracy in cattle face recognition, its robustness and generalization capabilities remain under-validated in complex pig face recognition scenarios. Xu et al. [11] proposed EHV-YOLO, a lightweight pig face recognition method based on EMobileNet and Horizontal-Vertical Attention Mechanism (HVAM). This approach achieved an average precision of 99.34% on the JD pig face dataset with only 0.97 M model parameters. However, this approach primarily focuses on single-pig face recognition. Further research is needed regarding its capability to identify multiple pigs simultaneously and recognize faces under growth-stage-related appearance variation. Marsot et al. [12] proposed an adaptive CNN-based pig face recognition method that automatically detects pig faces and eyes using a Haar feature cascade classifier, followed by recognition via a deep CNN. This approach achieved 83% accuracy on 320 test images from 10 pigs. However, it demonstrated insufficient robustness under complex backgrounds and lighting conditions, and its recognition capability for pigs at different growth stages was not thoroughly validated.

Guangbo Li et al. [13] proposed a rapid pig face recognition method based on an improved YOLOv3. This study introduced DenseBlock and an enhanced SPP unit to construct the YOLOv3_DB_SPP model, effectively enhancing feature extraction capabilities and detection accuracy, particularly demonstrating strong performance when detecting small target samples. However, the model still has room for improvement in localization accuracy, and detection speed decreases due to increased network complexity from these enhancements. Shuiqing Xu et al. [14] designed a pig face recognition method based on Trapezoidal Normalized Pixel Difference (T-NPD) features and Truncated Mean Attention Mechanism (TMAM). T-NPD features adapt to the unique shape of pig faces, improving detection accuracy; while TMAM further enhances classification accuracy by optimizing feature channel weights. However, this approach primarily targets single-pig-face recognition, and its performance and efficiency may face challenges when handling multiple pig faces in complex scenes. Masum Billah et al. [15] proposed a real-time goat face recognition method based on convolutional neural networks. This study employs a YOLO-based approach for goat face and facial feature point detection, utilizing a custom CNN model for identity recognition, achieving high accuracy. However, the method exhibits limited adaptability to multi-angle and multi-pose variations in goat faces during detection and recognition, and its generalization capability on large-scale datasets remains to be validated.

Ying Guo et al. [16] constructed a sheep face image dataset comprising 10 sheep breeds and proposed a knowledge distillation-based DT-YOLOv5s model for sheep breed recognition. This model enhances feature extraction capabilities and recognition accuracy by transferring knowledge from the teacher network YOLOv5x to the lightweight student network YOLOv5s, while maintaining fast inference speed. However, the study primarily focuses on sheep breed recognition. Its applicability and effectiveness for specific requirements in pig face recognition, such as identifying individuals at different growth stages, remain insufficiently validated. Hansen et al. [17] proposed a method for pig face recognition using convolutional neural networks (CNNs), achieving 96.7% accuracy. However, this approach primarily targets individual pig face images and demands substantial dataset scale and diversity. Ma et al. [18] proposed a lightweight model based on an improved YOLOv4 and MobileNet-v3 architecture. By incorporating deep separable convolutions and CBAM attention mechanisms, it effectively reduces parameter complexity while maintaining high recognition accuracy, thereby meeting practical demands for speed and cost efficiency. However, these approaches are primarily suited for closed-set scenarios and struggle to adapt to recognition tasks involving unseen individuals. To address this, Wang et al. [19] combined residual networks with the NAM attention mechanism to construct the ResNAM feature extraction network. They further introduced metric learning and an open-set recognition framework, enabling the model to achieve 95.28% recognition accuracy on unseen individuals. Meanwhile, Wang et al. [20] proposed a cascaded detection-and-recognition network architecture. This approach automatically removes complex backgrounds through a detection network, then combines it with a recognition network optimized by residual modules. It maintains high accuracy ranging from 97.66% to 99.38% under multi-angle and complex environmental conditions, and further developed an automated application system. Guo et al. [21] introduced a lightweight pig face recognition network (RKNet-HAM) integrating a fourth-order Runge–Kutta method with a hybrid attention mechanism. This approach achieved 99.26% recognition accuracy even under low-resolution image conditions, with a model size of only 1.52 MB, demonstrating strong potential for embedded deployment. However, this approach is limited to closed-set recognition, struggling to handle new individuals not present in the training dataset, thereby restricting its adaptability in real-world farming scenarios.

Although existing studies have achieved encouraging progress in pig face detection and recognition, most methods are still developed and validated under relatively constrained conditions, and their adaptability to growth-stage adaptive pig face recognition in practical farming environments remains insufficient. In particular, challenges related to growth-stage variation, environmental interference, and systematic performance evaluation have not been fully addressed.

However, several research gaps remain in current pig face recognition studies. First, most existing studies focus on pig identification under relatively fixed growth conditions, while insufficient attention has been paid to recognition consistency under growth-stage-induced morphological variation and feature distribution shifts, which limits the robustness of existing models for growth-stage adaptive pig face recognition. Second, although existing detection models have achieved promising performance, their adaptability remains limited under practical challenges such as scale inconsistency, occlusion interference, and complex farming backgrounds, which may reduce identity discrimination performance in real-world scenarios. Third, current studies mainly rely on conventional detection metrics, while identity-level evaluation for pig face recognition remains insufficient, limiting the scientific rigor of performance assessment.

To address these limitations, this study proposes a DESA-YOLO framework for growth-stage adaptive pig face recognition. In this study, “growth-stage adaptive” refers to improving recognition robustness under heterogeneous stage-related appearance variations, rather than strict longitudinal identity tracking of the same pig throughout the complete growth cycle. Under this definition, the task is formulated as a multi-class object detection problem for individual pig face recognition, where each pig identity is treated as an independent category and each sample is jointly annotated with localization and identity labels.

The main contributions of this work are summarized as follows:

(1) To improve individual pig recognition robustness under growth-stage-related appearance variation, a growth-stage adaptive pig face recognition framework based on a multi-class object detection paradigm is proposed, enabling joint pig face localization and identity recognition under heterogeneous growth-stage conditions.
(2) To overcome the limitations of conventional detectors in handling morphology variation, feature instability, occlusion interference, and multi-scale inconsistency, a collaborative optimization strategy integrating DualConv, EMA, SEAM, and ASFF is designed to enhance feature extraction, representation stability, discriminative attention, and adaptive feature fusion.
(3) To address the lack of systematic evaluation in existing studies, comprehensive experiments including comparative analysis, ablation studies, inference efficiency analysis, and identity-level evaluation are conducted to validate the effectiveness, robustness, and practical applicability of the proposed method.

These contributions establish a challenge-driven solution framework and provide support for improving individual pig recognition under practical intelligent farming conditions.

## 2. Materials and Methods

### 2.1. Data Collection

**Figure 1:**
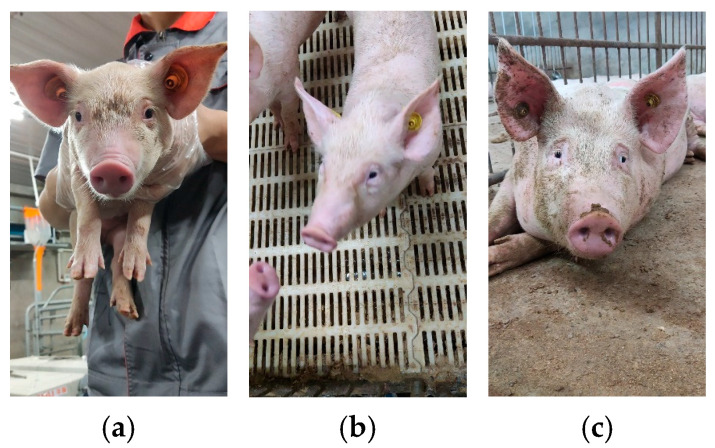
Representative pig face samples collected from three growth stages: (**a**) suckling stage, (**b**) weaning stage, and (**c**) fattening stage.

The video data for this study was collected by team members at the Tianjin Ninghe Original Breeding Pig Farm, Tianjin, China (Latitude: 39.44° N, Longitude: 117.64° E). Data collection took place from 30 July to 11 August 2024. A total of 144 breeding pigs from two breeds—Large White and Landrace—were sampled across three distinct growth stages. The three growth stages encompassed the suckling period, weaning period, and fattening period, with video footage captured for 48 breeding pigs per stage. Mobile phones were used for filming. Images were recorded at a resolution of 1920 × 1080 with a frame rate (fps) set to 30 to minimize blurring caused by increased pig movement due to camera shyness. Ultimately, videos of 144 pigs were captured, each lasting approximately 1–2 min and saved in MP4 format. Ear tag numbers served as individual IDs: suckling pigs (0–1 month old), weaning pigs (approximately 1–3 months old), and fattening pigs (3–6 months old). Figure 1 shows partial datasets collected from the three distinct growth stages [22]: a represents the suckling stage, b the weaning stage, and c the fattening stage. Significant facial differences are observable among the three growth stages.

Unlike conventional binary pig face detection datasets that only distinguish pig face regions from the background, the dataset constructed in this study was designed for individual pig face recognition under a multi-class object detection framework. Specifically, each pig face instance was annotated with both a bounding box and a unique identity label, where each individual pig was treated as an independent category, enabling joint pig face localization and identity recognition. During annotation, each pig face instance was assigned one bounding box and one corresponding identity label in YOLO format, following a unified rule that localization and identity labeling were jointly completed for every sample. Identity categories correspond to individual pigs rather than growth stages.

It should be noted that this dataset is not based on continuous longitudinal tracking of the same pigs throughout the complete growth cycle. Rather, it was constructed to evaluate model robustness under representative growth-stage-related appearance variations. Therefore, the implemented task is formulated as individual pig face recognition under growth-stage variation, rather than a binary pig face detection task or a growth-stage classification task.

The dataset contains 144 identity categories spanning three growth stages. This dataset construction logic, including task definition, unified annotation rules, and identity-based category design, is consistent with the objective of growth-stage adaptive pig face recognition and provides the basis for subsequent model training and evaluation.

### 2.2. Data Preprocessing and Dataset Construction

#### 2.2.1. Data Preprocessing

After acquiring pig face videos in natural settings, the captured video data was processed at an appropriate frame rate to extract raw pig face images. Each video was decomposed using OpenCV (version 4.9.0) [23] with a setting of 100 frames. The captured images contained some invalid data, such as occlusions from other pigs in the pen, blurred samples, or samples showing only partial faces. Additionally, adjacent images exhibited excessive similarity or near-duplication, potentially causing model overfitting. Therefore, data filtering was necessary to remove non-compliant samples. To address these issues arising during filming, manual selection was employed for processing. To ensure training consistency, the number of pig face data points per pig was kept consistent across each stage. The final raw pig face dataset comprised 20 images per pig, selected from different angles with significant variation. Across the three periods, this yielded a total of 2880 images—960 images per period. Data annotation was performed using LabelImg(version 1.8.6), directly converting data into YOLO format. This annotation process added bounding boxes and generated corresponding label files for the pig face regions within each image. This file primarily contains the four vertex coordinates of the rectangular region to be annotated on the pig’s face and the corresponding pig ID within that region. When annotating pig faces, the area from the outer edge of the eye socket to the outer edge of the nose was selected as the marking range. Since pig ears move freely, increasing the difficulty of feature extraction, the ear region was not annotated during the process. The annotation name is labeled as Growth Period-Ear Tag Number. for example, “suckling-016601” indicates sample 016601 in the suckling stage.

#### 2.2.2. Dataset Construction

**Figure 2:**
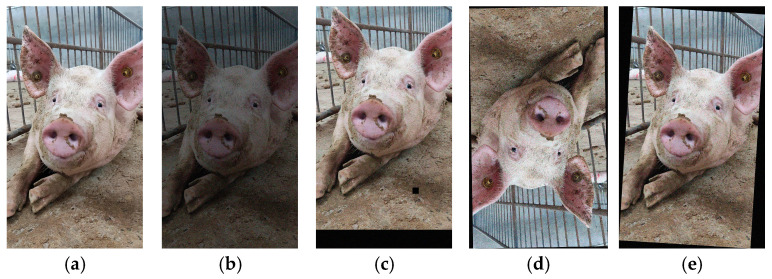
Demonstration of data augmentation techniques, (**a**) Original, (**b**) brightness variation, (**c**) translation, (**d**) flipping, (**e**) rotation.

The total number of original images in this dataset is 2880. The preprocessed dataset is divided into training, validation, and test sets at a ratio of 8:1:1. Under farm conditions, pigs exhibit frequent movement. Direct facial recognition often encounters challenges such as variations in the position, orientation, and size of pig faces within images, changes in ambient lighting conditions, and varying degrees of blurring. Failure to adequately account for these factors during dataset construction can adversely affect recognition results. Deep learning-based object detection models are generated through training on extensive image datasets. Therefore, data augmentation is applied to the 2880 images in the training and validation sets [24]. This includes brightness enhancement and reduction, horizontal flipping, vertical flipping, multi-angle rotation, Gaussian noise addition, and mosaic data augmentation. The data augmentation process is as follows: To specifically address such issues and enhance the model’s generalization capability, this paper employs four randomly selected data augmentation methods [25]. The dataset is expanded to four times its original size. The data augmentation effect on the same image is shown in Figure 2.

The augmented dataset comprises 14,400 images, forming the pigface dataset. Each individual stage contains 960 original images, with 4800 augmented images per stage. Detailed statistics of the augmented dataset are presented in Table 1. The augmented dataset is named “pig face”; the experiments described below are based on this dataset.

### 2.3. Model Architecture of DESA-YOLO

**Figure 3:**
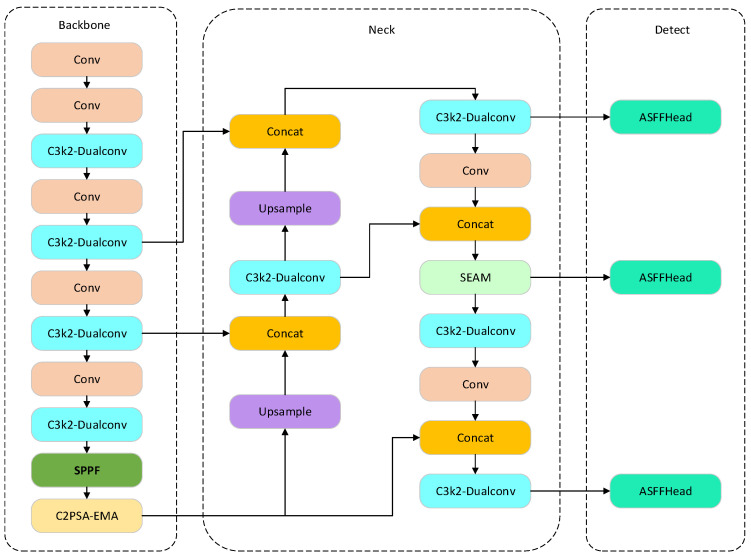
DESA-YOLO Network Architecture Diagram.

To improve detection accuracy and growth-stage adaptability in pig face recognition, this study develops an improved DESA-YOLO model based on the YOLO11 framework [26]. The model maintains the lightweight architecture and fast detection speed of YOLO11, while optimizing its feature extraction and feature fusion modules. The overall architecture of DESA-YOLO consists of an input layer, a backbone network, a feature enhancement network, and a detection head, as illustrated in Figure 3. In the input layer, the input images undergo adaptive anchor matching and dynamic image scaling to improve adaptability to targets of different sizes. In the backbone network, the original C3k2 module is replaced with a DualConv module [27] to reduce information redundancy and enhance feature representation capabilities. In the feature enhancement stage, the Efficient Multi-Scale Attention (EMA) module [28] and the Split Enhanced Attention Mechanism (SEAM) [29] are incorporated to enhance the model’s feature extraction capability for key regions. In the detection head, the original YOLO11 detection module is replaced with an Adaptive Spatial Feature Fusion (ASFF) [30] head to achieve adaptive multi-scale feature fusion and improve detection capability for pig faces of varying scales.

Growth-stage adaptive pig face recognition differs from conventional object detection because pig facial features exhibit significant variations in scale, morphology, and feature distribution under growth-stage-related appearance variation. As shown in the stage-specific experimental analysis, piglets, weaning pigs, and fattening pigs present differences in facial size, contour structure, and discriminative texture patterns, while practical farming environments further introduce occlusion and background interference. These factors jointly increase the difficulty of maintaining robust feature extraction and identity recognition under heterogeneous growth-stage conditions.

Therefore, the integration of DualConv, EMA, SEAM, and ASFF is not a simple accumulation of existing structures, but a challenge-driven collaborative design. Specifically, DualConv improves the representation of stage-dependent facial morphology, EMA enhances discriminative feature representation through adaptive attention weighting, SEAM enhances attention to discriminative facial regions under occlusion, and ASFF improves adaptive fusion of multi-scale features caused by facial size variation. These modules correspond to different challenges induced by growth-stage-related appearance variation and operate at complementary levels of the network, including feature extraction, representation stabilization, attention refinement, and detection fusion.

Accordingly, these modules form a collaborative optimization framework for growth-stage adaptive pig face recognition, rather than a simple combination of independent improvements. This design rationale is further supported by the ablation results in Section 3.3, where each module contributes incremental performance gains and the complete framework achieves the best overall recognition performance.

The improved model is capable of extracting richer deep semantic features and exhibits stronger resistance to occlusion and better focus on local discriminative features. The main improvements of DESA-YOLO focus on enhancing detection accuracy and feature representation while maintaining detection speed, making it suitable for pig face recognition in complex environments.

#### 2.3.1. DualConv Module Structure

Although the C3k2 structure in the YOLO11 backbone has strong feature extraction capabilities, its stacked convolution form is inefficient in handling redundant features and may lead to feature redundancy. To reduce computational redundancy and enhance feature representation, this study replaces the original C3k2 module with the DualConv module. The DualConv module consists of two parallel convolution branches: a 3 × 3 convolution branch and a 1 × 1 convolution branch. The 3 × 3 convolution branch is used to extract local spatial features and expand the receptive field, while the 1 × 1 convolution branch performs channel compression and feature fusion to reduce redundant information [27]. The outputs of the two branches are fused through concatenation, enabling the complementarity of shallow and deep information and improving the refinement of feature representation.

**Figure 4:**
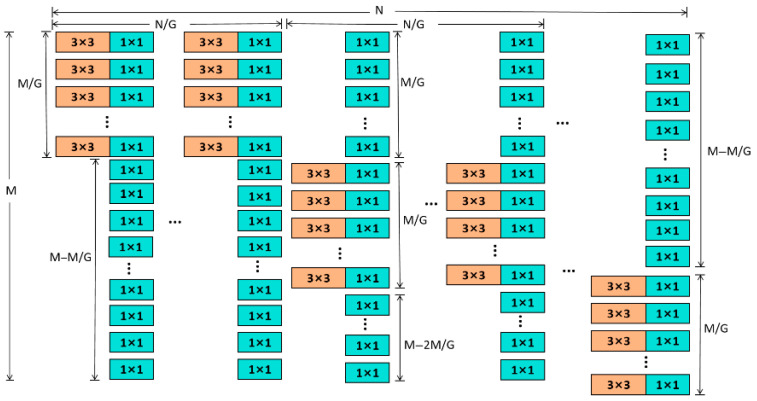
Dualconv Network Structure Diagram.

The DualConv feature fusion process can be expressed as:

(1) $$F_{out}=Concat(Conv_{3\times 3}(X),Conv_{1\times 1}(X))$$

where X denotes the input feature map, $Conv_{3\times 3}$ extracts local spatial features, $Conv_{1\times 1}$ performs channel compression and feature fusion, and Concat(⋅) denotes feature concatenation. The DualConv module effectively improves feature extraction efficiency while maintaining low computational cost. The structure of the DualConv module is shown in Figure 4.

#### 2.3.2. Efficient Multi-Scale Attention (EMA) Module

In object detection tasks, feature extraction is often affected by noise interference, scale variation, and background complexity, which may lead to insufficient focus on discriminative regions and unstable feature representation. This problem is more severe in complex pig face recognition scenarios involving partial occlusion and growth-stage-related appearance variation. To improve the effectiveness of feature extraction, this study incorporates an Efficient Multi-Scale Attention (EMA) module into the network.

The EMA module enhances feature representation by modeling both spatial dependencies and channel interactions through average pooling, feature concatenation, and adaptive attention weighting. First, the input feature map is aggregated through average pooling to capture global contextual information:

(2) S=AvgPool(F)

where F denotes the input feature map and S represents the pooled feature representation.

Subsequently, the pooled features are concatenated with the original feature map and transformed through convolution and sigmoid activation to generate adaptive attention weights:

(3) A=σ(Conv(Concat(S,F)))

Finally, the attention weights are applied to the input feature map for adaptive feature enhancement:

(4) $$F^{\prime }=A⊙F$$

where A denotes the attention weights, σ represents the Sigmoid activation function, and ⊙ denotes element-wise multiplication.

**Figure 5:**
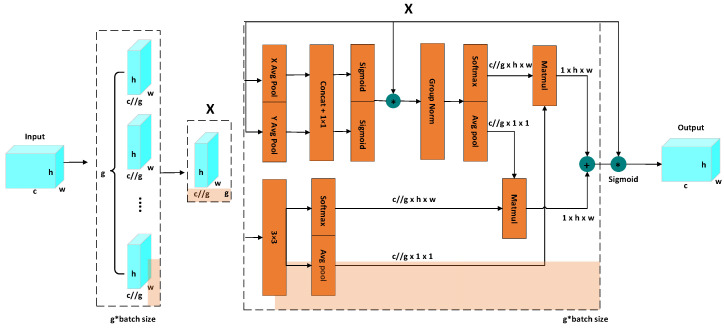
EMA Network Architecture Diagram.

Through this adaptive weighting mechanism, the EMA module enables the network to focus more effectively on discriminative pig facial regions while suppressing irrelevant background responses, thereby improving robustness under scale variation and partial occlusion. In addition, the module introduces limited computational overhead while enhancing multi-scale feature interaction, making it suitable for integration into lightweight YOLO-based detection networks. Unlike DualConv, which mainly improves local feature extraction, the EMA module focuses on enhancing feature representation through adaptive attention weighting and contributes to stabilizing discriminative feature learning under growth-stage-related appearance variation. Its individual contribution is further quantitatively validated in the ablation experiments in Section 3.3. The network architecture diagram of EMA is shown in Figure 5. where “*” denotes element-wise multiplication used for feature weighting, and “+” denotes the feature fusion operation.

#### 2.3.3. SEAM (Spatially Enhanced Attention Module)

**Figure 6:**
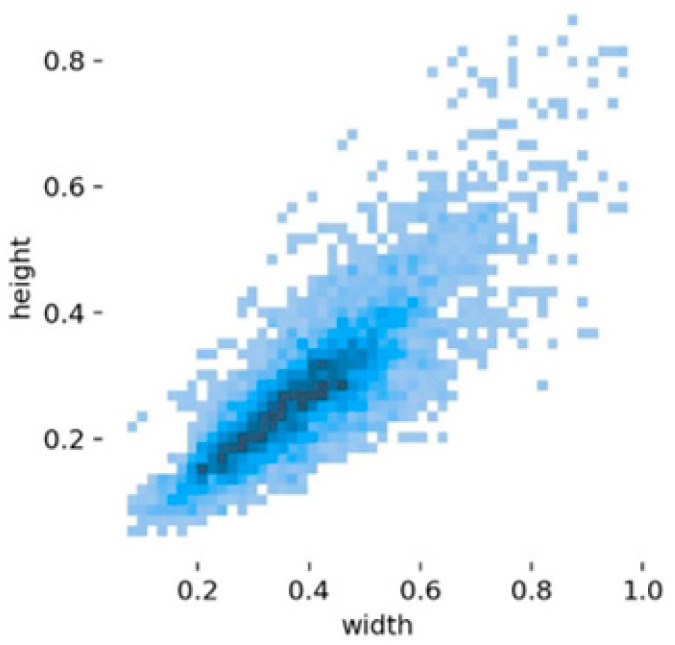
Data Distribution in the Dataset.

**Figure 7:**
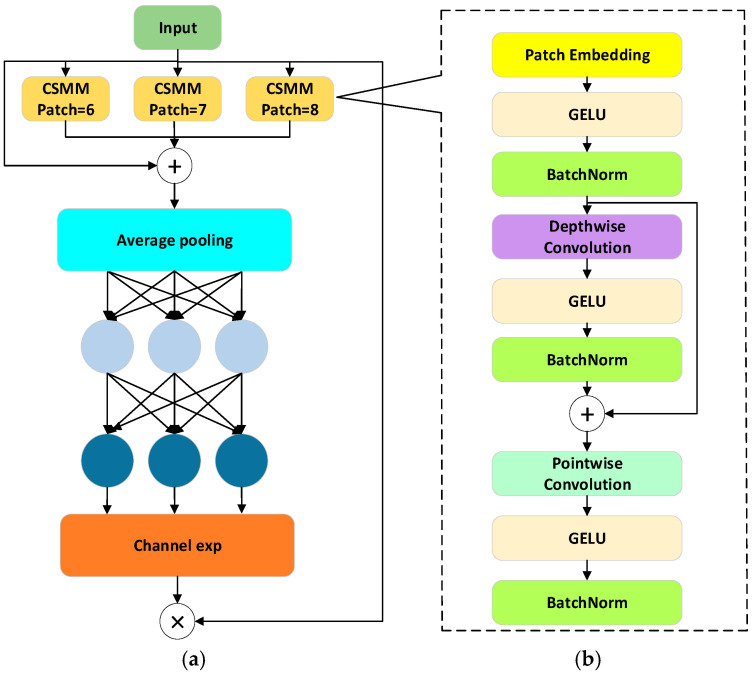
SEAM Network Architecture Diagram, (**a**) The Overall Architecture of SEAM, (**b**) Detailed Structure of the CSMM Module.

As shown in Figure 6, the distribution of target bounding box widths and heights indicates that the dataset is dominated by small and medium-sized targets, with a slightly higher proportion of medium-scale targets, while large targets are relatively scarce. Since small targets are prone to feature loss during representation, we introduce the SEAM (Spatially Enhanced Attention Module) attention mechanism into the neck of the network to enhance feature representation capability for small and partially occluded targets [31]. The SEAM module combines depthwise separable convolution with residual connections to reduce the number of parameters while emphasizing the importance of different channels. To overcome the limitation of depthwise separable convolution, which lacks modeling of inter-channel relationships, SEAM introduces a 1 × 1 convolution after feature extraction for channel fusion and employs a two-layer fully connected network to establish cross-channel dependencies, thereby strengthening inter-channel correlations. The attention weighting process can be expressed as:

(5) $$A=\sigma (W_{2}\delta (W_{1}F))$$

(6) $$F^{\prime }=A⊙F$$

where F denotes the input feature map, $W_{1}$ and $W_{2}$ are learnable weights, δ denotes the ReLU activation function, σ denotes the Sigmoid activation, and ⊙ represents element-wise multiplication. This design maintains lightweight computation while effectively modeling feature differences between occluded and non-occluded regions, thereby improving the model’s robustness to small and occluded targets. The SEAM architecture is illustrated in Figure 7, where the left part shows the overall structure consisting of three CSMM modules with different patch sizes (patch-6, patch-7, patch-8). The outputs of these modules are average pooled, followed by channel expansion (Channel exp), and then multiplied to produce enhanced feature representations. The right part shows the detailed structure of the CSMM module, which leverages multi-scale features through different patch sizes and employs depthwise separable convolution to learn correlations between spatial dimensions and channels.

#### 2.3.4. ASFF (Adaptive Spatial Feature Fusion)

**Figure 8:**
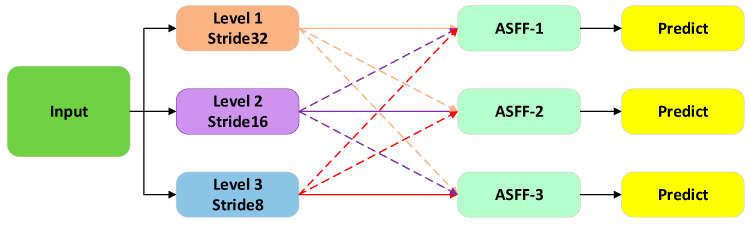
ASFF Network Architecture Diagram.

Finally, we replaced the original Detect head of YOLO11 with the ASFF (Adaptive Spatial Feature Fusion) module [32] to address the inconsistency between features at different scales. The structure of the ASFF module is shown in Figure 8. The core innovation of ASFF lies in its ability to adaptively fuse spatial features, effectively filtering conflicting information and enhancing the scale invariance of the model. Compared with conventional detection heads, ASFF can be trained directly through backpropagation without depending on the original model architecture, while introducing minimal computational overhead. The adaptive feature fusion process is defined as:

(7) $$F_{fusion}=\lambda_{1}F_{1}+\lambda_{2}F_{2}+\lambda_{3}F_{3}$$

Subject to:

(8) $$\lambda_{1}+\lambda_{2}+\lambda_{3}=1$$

where $F_{1},F_{2},F_{3}$ denote features at different scales, and $\lambda_{i}$ are adaptive learnable fusion weights.

This improvement not only enhances the model’s performance in multi-scale object detection but also strengthens its feature fusion capability, providing higher accuracy and robustness for object recognition in complex scenarios.

### 2.4. Experimental Settings

The experimental environment was based on a 64-bit Windows 10 operating system, using Anaconda3 as the software platform and PyCharm (version 2023.1.4) as the integrated development environment. The computer was equipped with 64 GB of RAM and utilized an NVIDIA RTX A5000 GPU for accelerated image processing. The specific model hyperparameter settings are shown in Table 2.

### 2.5. Evaluation Metrics

Under this multi-class detection setting, Precision, Recall, mAP, and F1-score evaluate both localization performance and identity classification correctness. To comprehensively evaluate the model performance of the YOLO11 model under growth-stage-related appearance variation, this experiment employed Precision, Recall, mean Average Precision (mAP), and F1-score to measure model performance. The calculation formulas for the evaluation metrics are as follows:

(9) $$Precision=\frac{TP}{TP+FP}$$

(10) $$Recall=\frac{TP}{TP+FN}$$

(11) $$mAP=\frac{\sum_{1}^{N}\int_{0}^{1}P(R)dR}{N}$$

(12) $$F1=\frac{2\times P\times R}{P+R}\times 100\%$$

where TP (True Positive) denotes the number of positive samples correctly classified as positive, TN (True Negative) denotes the number of negative samples correctly classified as negative, FP (False Positive) denotes the number of negative samples incorrectly classified as positive, and FN (False Negative) denotes the number of positive samples incorrectly classified as negative. AP is the area under the Precision–Recall (P–R) curve, and mAP is the mean value of AP across all categories. N denotes the number of classes in the test samples.

In addition to detection accuracy metrics, model efficiency and deployment feasibility were further evaluated using the number of parameters (Params), model size, computational complexity (GFLOPs), inference speed (FPS), and inference latency. GFLOPs represents the floating-point operations required for a single forward inference and is used to measure computational cost. FPS denotes the number of images processed per second and was calculated as:

(13) $$FPS=\frac{N_{f}}{T}$$

where $N_{f}$ is the number of processed images and T is the total inference time. These efficiency metrics were introduced to comprehensively evaluate not only detection performance but also the real-time applicability and deployment feasibility of the proposed model in intelligent farming scenarios.

## 3. Results

### 3.1. Comparison with Different Models

To objectively verify the effectiveness of the proposed method, we compared the improved DESA-YOLO model with YOLOv5 [33], YOLOv8 [34], YOLOv10 [35], YOLO11 [36], YOLO12 [37], and Faster-RCNN [38], as well as recent state-of-the-art transformer-based and hybrid detectors, including RT-DETR-R18 [39], Gold-YOLO-N [40], and DINO-R50 [41]. The comparison results are presented in Table 3. As shown in Table 3, the proposed DESA-YOLO achieved the best overall detection performance among all compared methods, reaching 92.8% precision, 88.8% recall, 93.7% mAP@0.5, and 90.7% F1-score. Compared with YOLO11, DESA-YOLO improved mAP by 3.0% and F1-score by 4.9%, while achieving gains of 6.0%, 6.2%, 7.7%, 6.3%, and 8.4% in mAP over YOLOv5, YOLOv8, YOLOv10, YOLO12, and Faster-RCNN, respectively. In addition, DESA-YOLO also outperformed recent state-of-the-art models, improving mAP by 1.9%, 1.3%, and 0.9% over RT-DETR-R18, Gold-YOLO-N, and DINO-R50, respectively, demonstrating the superiority of the proposed architecture in pig face recognition tasks.

In terms of computational efficiency and deployment feasibility, DESA-YOLO maintained a favorable balance between accuracy and complexity. Although the proposed model increased computational cost to 9.4 GFLOPs compared with YOLO11 (6.8 GFLOPs), it still achieved real-time inference at 109 FPS, with only a slight reduction in speed relative to YOLO11 (124 FPS). Moreover, DESA-YOLO exhibited substantially lower computational complexity and higher inference speed than heavier transformer-based methods such as DINO-R50, while maintaining higher detection accuracy. These results indicate that the proposed method not only improves recognition performance but also preserves practical applicability for real-time intelligent farming deployment.

**Figure 9:**
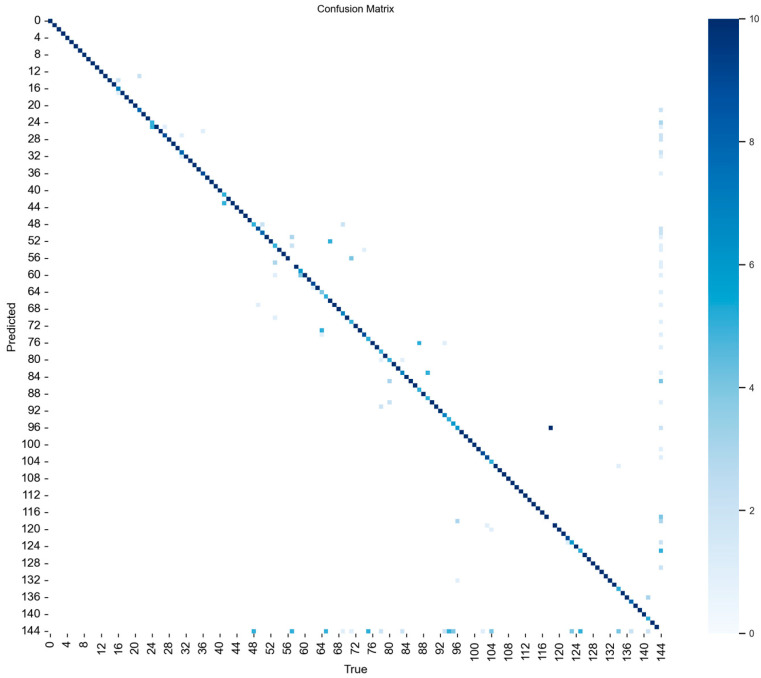
Confusion matrix of pig identity recognition results for 144 individual pigs.

To further assess the identity discrimination capability of the proposed model beyond detection-based metrics, an identity-level evaluation was conducted using a confusion matrix, as shown in Figure 9. The confusion matrix exhibits a strong concentration along the main diagonal, indicating that most pig identities were correctly classified. Meanwhile, off-diagonal elements are sparse and primarily distributed among a limited number of visually similar individuals, suggesting that inter-individual confusion is relatively low.

This result provides additional evidence that the proposed DESA-YOLO improves not only detection performance in terms of precision, recall, and mAP, but also maintains reliable discrimination among individual pigs. In particular, the limited confusion observed among some identities is mainly attributable to similar facial appearance, stage-dependent morphological changes, and partial occlusion in practical farming environments, which is consistent with the challenges discussed in this study. In addition, the confusion matrix provides identity-level evidence that the proposed method reduces inter-individual misclassification. The confusion matrix complements conventional detection metrics by providing additional identity-level evidence of reduced inter-individual misclassification.

### 3.2. Comparison of Training Results Across Different Growth Stages

To further evaluate the effectiveness of the DESA-YOLO model in addressing growth-related variations in pigs, the dataset was divided according to growth stages, and the baseline YOLO11 model was compared with the improved model in terms of key performance metrics including Precision, Recall, and mean Average Precision (mAP). Detailed results are shown in Table 4.

Analysis of the results in Table 4 shows that the DESA-YOLO model outperforms the YOLO11 baseline model in most cases, achieving a significant performance breakthrough particularly during the weaning stage, which is the most challenging to recognize. First, during the suckling and fattening stages, both models achieved high performance (mAP > 97.8% and mAP > 93.0%), indicating that pig facial features are relatively stable and easier to recognize in these two stages. DESA-YOLO achieved significant improvements in Precision at these two stages (+1.4% and +4.7% respectively), demonstrating that the improved model identifies positive samples more accurately and effectively reduces false detections. Although Recall shows a slight decrease (−0.2% and −0.4% respectively), it can be considered statistically unchanged, and the consistent improvement in mAP confirms the overall superiority of DESA-YOLO. The most notable improvement appears in the weaning stage. This stage is a critical transitional period in pig growth, during which facial morphology changes dramatically and individual differences increase, resulting in a significant performance decline of the YOLO11 baseline (mAP dropping to 83.0% and Recall to only 70.1%). In contrast, the DESA-YOLO model demonstrates strong adaptability at this stage, with all metrics improving significantly, including a 12.6 percentage point increase in Recall and a 7.4 percentage point increase in mAP. These results strongly demonstrate that the proposed multi-scale feature fusion and attention mechanisms effectively enhance the model’s ability to capture scale-varying and low-quality features, significantly reducing missed detections and addressing the core weakness of unstable recognition in the baseline model during transitional growth stages.

### 3.3. Ablation Experiments

To ensure the reliability of the experiments, four sets of ablation experiments were designed. All experiments were conducted using the same equipment, identical environment settings, and the same dataset for training and validation. Table 5 presents the results of five experiments incorporating four different improvement strategies. Experiment E0 serves as the baseline without any improvement, while experiment EP incorporates all the improvement strategies. In the improved model configurations, E1 replaces C3K2 with DualConv, E2 replaces C2PSA with EMA, E3 adds SEAM, and EP replaces the detection head with ASFFHead.

As shown in Table 5, all improved models outperform the baseline E0 in terms of mAP, demonstrating the effectiveness of each proposed module. Compared with E0, E1 improves mAP from 90.7% to 92.4%, indicating that DualConv enhances feature extraction capability. Based on E1, E2 further increases mAP to 93.0%, showing that the introduction of EMA improves feature representation and target discrimination. After incorporating SEAM, E3 increases mAP to 93.3%, corresponding to a 2.6% improvement over the baseline. Finally, the complete EP model achieves the best overall performance, reaching 93.7% mAP and 90.7% F1-score, representing a 3.0% improvement in mAP compared with E0.

In addition to accuracy improvements, the ablation results also reveal the computational impact of each module. As modules are progressively introduced, computational complexity increases from 6.8 GFLOPs in E0 to 9.4 GFLOPs in EP, while inference speed decreases moderately from 124 FPS to 109 FPS. Despite this increase in computational cost, the reduction in inference speed is limited, indicating that the proposed modules introduce acceptable overhead while maintaining real-time performance. This demonstrates that the performance gains are achieved through a favorable balance between detection accuracy and computational efficiency.

Moreover, parameter size increases from 2.6 M in E0 to 3.9 M in EP, while the model size grows from 5.6 MB to 8.3 MB, further confirming that the proposed improvements preserve lightweight deployment characteristics. Therefore, the complete EP model achieves the best combination of accuracy, efficiency, and deployment feasibility.

**Figure 10:**
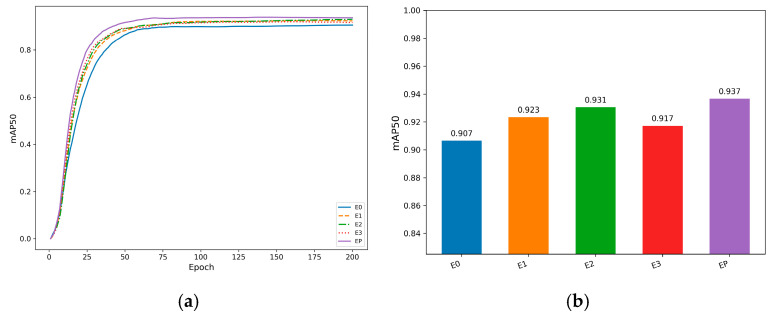
Ablation analysis of module contributions: (**a**) mAP trends under progressive module integration; (**b**) comparative performance gains of different model variants.

The mAP comparison curve and bar chart of the models are shown in Figure 10, which further demonstrates that EP achieves the optimal overall improvement effect.

## 4. Discussion

### 4.1. Comparative Analysis of Individual Detection Performance

**Figure 11:**
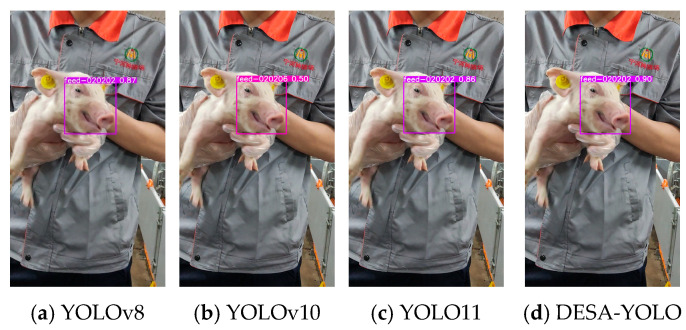
Detection result comparison of YOLOv8, YOLOv10, YOLO11, and DESA-YOLO on representative suckling-stage samples.

**Figure 12:**
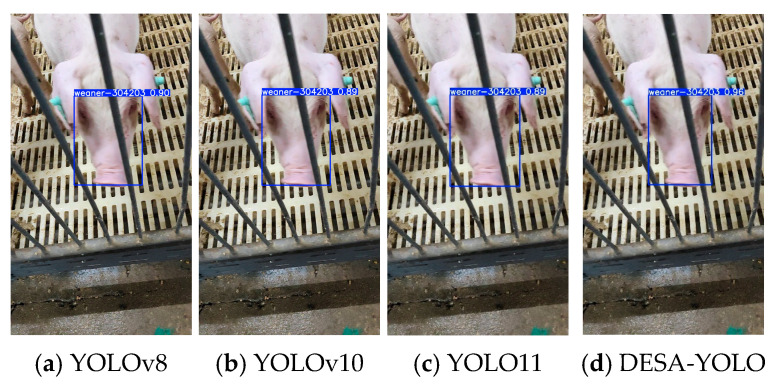
Detection result comparison of YOLOv8, YOLOv10, YOLO11, and DESA-YOLO on representative weaning-stage samples.

**Figure 13:**
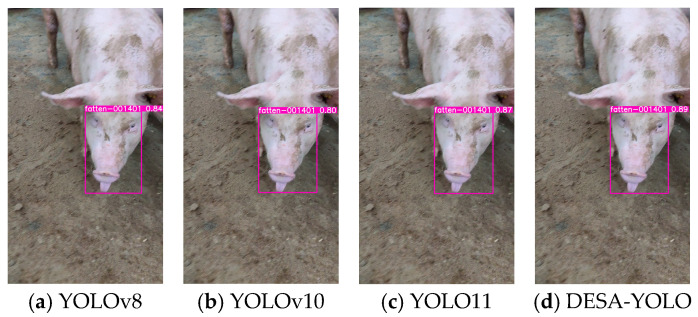
Detection result comparison of YOLOv8, YOLOv10, YOLO11, and DESA-YOLO on representative fattening-stage samples.

To intuitively demonstrate the performance of the proposed DESA-YOLO algorithm in practical detection tasks, sample images were randomly selected from the test set, and the individual detection results of different models across three typical growth stages—suckling, weaning, and fattening—were compared, as shown in Figure 11, Figure 12 and Figure 13. The text above each bounding box indicates the identified pig individual ID, and the numerical value represents the prediction confidence.

The comparative results show that YOLOv8, YOLOv10, and YOLO11 are capable of individual detection in certain cases, but they commonly suffer from issues such as bounding box misalignment, low confidence scores, and insufficient sensitivity to small targets. This problem is particularly pronounced in weaning-stage samples, where small facial regions and frequent occlusion lead to frequent missed detections or false detections by mainstream models. In fattening-stage samples, the large body size and diverse postures of pigs result in unstable bounding boxes in some models, reducing detection accuracy.

In contrast, DESA-YOLO exhibits higher detection accuracy and robustness across all three growth stages. Its bounding box positions are more precise, prediction confidence scores are significantly higher than those of the comparison models, and it maintains strong model performance for small targets and complex scenarios such as partial facial occlusion and dense individual distributions. These results indicate that the proposed improvements effectively alleviate the instability issues commonly observed in mainstream models for pig individual detection, thereby enhancing overall detection performance and applicability.

### 4.2. Comparative Analysis of Heatmap-Based Detection Results

**Figure 14:**
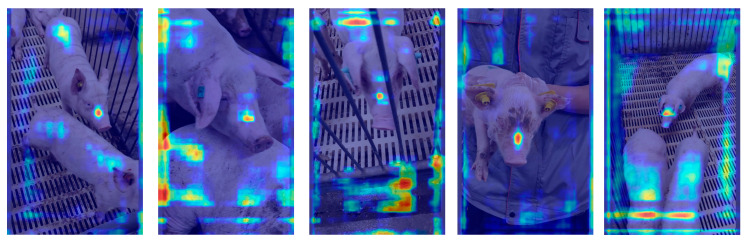
Grad-CAM visualization of discriminative feature responses produced by the baseline YOLO11 model.

**Figure 15:**
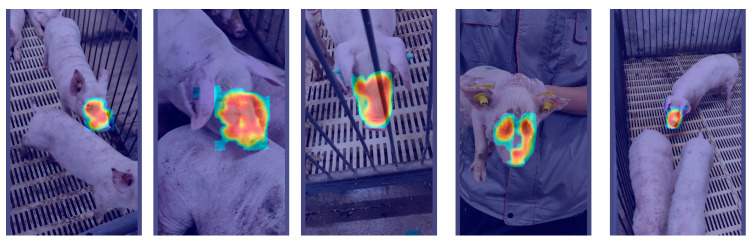
Grad-CAM visualization of discriminative feature responses produced by the proposed DESA-YOLO model.

To further verify the effectiveness of the proposed DESA-YOLO model in pig face individual recognition, we performed a comparative analysis of Grad-CAM [42] heatmap distributions before and after model improvement, as shown in Figure 14 and Figure 15. As shown in the figures, the original YOLO network exhibits scattered attention in some samples, with activation regions not only covering the pig’s face but also extending to the ears, body, and even background fence areas. This attention drift causes the model to be affected by background interference during feature extraction, thereby reducing its ability to identify key individual features and resulting in unstable performance across different growth stages.

In contrast, after incorporating the C3k2-DualConv, EMA, SEAM, and ASFF Head modules, the proposed DESA-YOLO model exhibits a much more focused and discriminative activation pattern in its attention distribution. Specifically, the model consistently focuses on key regions of the pig’s face, such as the nose, eyes, and forehead, while activation in background and non-discriminative regions is significantly reduced. This indicates that the improved network not only enhances its ability to capture local details during feature extraction but also improves robustness in complex environments through multi-scale feature fusion and semantic enhancement mechanisms.

Moreover, pigs at different growth stages exhibit significant variations in appearance, and traditional models are prone to attention shifts due to scale and pose variations. However, the heatmap results demonstrate that DESA-YOLO improves growth-stage adaptability, enabling the model to accurately focus on discriminative regions across multiple growth stages, including piglets, weaning pigs, and fattening pigs.

The Grad-CAM visualization is consistent with quantitative results, showing that DESA-YOLO produces more concentrated activations on discriminative facial regions while suppressing irrelevant background responses. This indicates that the proposed modules improve feature representation, attention allocation, and multi-scale fusion, thereby enhancing robustness across different growth stages.

### 4.3. Limitations and Generalization Analysis

Although this study focuses on pig face recognition under growth-stage variation, the constructed dataset is not based on longitudinal tracking of the same individuals throughout the complete growth cycle from suckling to fattening stages. Therefore, the present study does not fully validate continuous identity matching of the same pig across the entire developmental process in a strict longitudinal sense.

Instead, the objective of this study is to evaluate model robustness for individual pig recognition under representative growth-stage-related appearance variations, including differences in facial morphology, scale, and feature distribution. Under this setting, growth-stage adaptive recognition refers to recognition under heterogeneous stage conditions rather than full life-cycle identity tracking.

In addition, the dataset was collected from a single farm and includes only two pig breeds, which may limit broader cross-farm generalization. Future work will incorporate multi-farm and multi-breed datasets to further evaluate model generalizability. benchmarking on public pig face datasets was not included in the current study and will be incorporated in future work.

## 5. Conclusions

This study proposes an improved YOLO11-based detection model, DESA-YOLO, to address the challenges in pig face individual recognition, such as significant growth-stage-related appearance variation, high similarity of appearance features, and interference from complex farming environments. The model incorporates C3k2-DualConv in the backbone network to enhance lightweight feature extraction, and the EMA module to improve multi-scale modeling efficiency; integrates the SEAM attention mechanism to enhance robustness against small targets and occlusions; and introduces the ASFF adaptive spatial feature fusion in the detection head to achieve dynamic fusion of multi-scale information. Through the collaborative optimization of multiple modules, DESA-YOLO achieves significant improvements in feature extraction and discriminative capability.

Experimental results demonstrate that DESA-YOLO outperforms comparison models in pig face individual recognition tasks across three growth stages: suckling, weaning, and fattening. Notably, during the challenging weaning stage, the model achieves an improvement of over 7 percentage points in mAP compared to the baseline YOLO11, significantly alleviating the performance degradation observed in traditional methods. Meanwhile, ablation experiments verify the positive contribution of each improvement module to overall performance, confirming the rationality and effectiveness of the proposed strategies. Furthermore, Grad-CAM visualization results confirm that DESA-YOLO consistently focuses on key discriminative regions of pig faces (such as the nose, eyes, and forehead), effectively reducing background interference and enhancing growth-stage adaptability.

Overall, the proposed DESA-YOLO model demonstrates advantages in Precision, Recall, F1-score, and mAP while balancing recognition speed and accuracy, providing a feasible solution for high-precision individual identification of pigs under heterogeneous growth-stage conditions. This study provides theoretical support and practical guidance for individual recognition in intelligent farming scenarios and lays the foundation for future applications in precision livestock management, disease monitoring, and behavioral analysis. However, the present study was conducted on a single-farm dataset with two breeds, and broader cross-farm generalization should be further validated in future work.

## Figures and Tables

**Table 1:** Detailed Information on the Dataset.

Growth Period	Before Enhancement	After Enhancement	Training	Validation	Test
Overall Phase	2880	14,400	11,520	1440	1440
suckling	960	4800	3840	480	480
Weaning	960	4800	3840	480	480
Fattening	960	4800	3840	480	480

**Table 2:** Model Experimental Parameter Settings.

Parameter	Value
Optimizer	SGD
Epochs	200
Batch	16
Images-size	640
Momentum	0.937
Initial learning rate	0.01
Workers	8
Weight learning rate	0.0005

**Table 3:** Comparison of Experimental Results of Different Models.

Model	Precision (%)	Recall (%)	mAP (%)	F1	Param/M	Model Size/MB	GFLOPs	FPS
YOLOv5	79.7	82.7	87.7	81.1	2.26	4.8	6.3	129
YOLOv8	83.2	82.2	87.5	82.6	2.76	5.8	7.1	121
YOLOv10	80.9	79.5	86	80.1	2.85	6.1	7.5	118
YOLO11	86.5	85.2	90.7	85.8	2.66	5.6	6.8	124
YOLO12	79.4	83.3	87.4	81.3	2.63	5.7	6.9	123
Faster-RCNN	82.5	83.2	85.3	82.8	41.6	107.8	42.7	29
RT-DETR	89.4	86.9	91.8	88.1	4.52	17.4	10.6	102
Gold-YOLO-N	90.7	87.5	92.4	89.1	4.83	18.6	11.3	114
DINO-R50	91.2	87.8	92.8	89.5	47.2	181.3	56.5	28
DESA-YOLO	92.8	88.8	93.7	90.7	3.96	8.3	9.4	109

**Table 4:** Comparison of Performance Metrics between YOLO11 and DESA-YOLO across Different Growth Stages.

Growth Period	Evaluation Metrics	YOLO11	DESA-YOLO	Increase
suckling	Precision (%)	94.4	95.8	+1.4
	Recall (%)	93.1	92.9	−0.2
	mAP (%)	97.8	98.7	+0.9
weaning	Precision (%)	85.9	89.7	+3.8
	Recall (%)	70.1	82.7	+12.6
	mAP (%)	83	90.4	+7.4
fattening	Precision (%)	90.1	94.8	+4.7
	Recall (%)	89.5	89.1	−0.4
	mAP (%)	93	94.6	+1.6

**Table 5:** Comparison of Model Performance during the Improvement Process.

Model	DualConv	EMA	SEAM	ASFFHead	Precision (%)	Recall (%)	mAP(%)	F1	Param/M	Model Size/MB	GFLOPs	FPS
E0					86.5	85.2	90.7	85.8	2.6	5.6	6.8	124
E1	√				87.4	87.6	92.4	87.6	2.6	5.6	7.4	120
E2	√	√			92.2	85.3	93	87.6	2.5	5.5	7.9	117
E3	√	√	√		93.3	85.4	93.3	89.1	2.6	5.5	8.5	113
EP	√	√	√	√	92.8	88.8	93.7	90.7	3.9	8.3	9.4	109

Note: “√” indicates that the corresponding module is included in the model configuration.

## Data Availability

The data that support the findings of this study are available from the corresponding author upon reasonable request.

## References

[1] Gómez Y., Stygar A.H., Boumans I.J.M.M., Bokkers E.A.M., Pedersen L.J., Niemi J.K., Pastell M., Manteca X., Llonch P. (2021). A Systematic Review on Validated Precision Livestock Farming Technologies for Pig Production and Its Potential to Assess Animal Welfare. Front. Vet. Sci..

[2] Lin W., He X., Li Z., Lyu E., Liu Y., Zeng Z., Huang J. (2026). PigInstance: An Efficient Pig Instance Segmentation Framework for Intelligent Pig Farming. Comput. Electron. Agric..

[3] He Y., Song K., Meng Q., Yan Y. (2020). An End-to-End Steel Surface Defect Detection Approach via Fusing Multiple Hierarchical Features. IEEE Trans. Instrum. Meas..

[4] Guo H., Meng Q., Cao D., Chen H., Liu J., Shang B. (2022). Vehicle Trajectory Prediction Method Coupled with Ego Vehicle Motion Trend Under Dual Attention Mechanism. IEEE Trans. Instrum. Meas..

[5] Zeng N., Wu P., Wang Z., Li H., Liu W., Liu X. (2022). A Small-Sized Object Detection Oriented Multi-Scale Feature Fusion Approach with Application to Defect Detection. IEEE Trans. Instrum. Meas..

[6] Xu X., Zhao M., Shi P., Ren R., He X., Wei X., Yang H. (2022). Crack Detection and Comparison Study Based on Faster R-CNN and Mask R-CNN. Sensors.

[7] Seo J., Sa J., Choi Y., Chung Y., Park D., Kim H. (2019). A YOLO-Based Separation of Touching-Pigs for Smart Pig Farm Applications. Proceedings of the 2019 21st International Conference on Advanced Communication Technology (ICACT), PyeongChang, Republic of Korea, 17–20 February 2019.

[8] Nie L., Li B., Jiao F., Shao J., Yang T., Liu Z. (2023). ASPP-YOLOv5: A Study on Constructing Pig Facial Expression Recognition for Heat Stress. Comput. Electron. Agric..

[9] Wang Z., Liu T. (2022). Two-Stage Method Based on Triplet Margin Loss for Pig Face Recognition. Comput. Electron. Agric..

[10] Li Z., Lei X., Liu S. (2022). A Lightweight Deep Learning Model for Cattle Face Recognition. Comput. Electron. Agric..

[11] Xu S., Zheng H., Tao S., Chai Y., He Q., Chen H. (2024). A Lightweight Pig Face Recognition Method Based on Efficient Mobile Network and Horizontal Vertical Attention Mechanism. IEEE Trans. Instrum. Meas..

[12] Marsot M., Mei J., Shan X., Ye L., Feng P., Yan X., Li C., Zhao Y. (2020). An Adaptive Pig Face Recognition Approach Using Convolutional Neural Networks. Comput. Electron. Agric..

[13] Li G., Jiao J., Shi G., Ma H., Gu L., Tao L. (2022). Fast Recognition of Pig Faces Based on Improved Yolov3. J. Phys. Conf. Ser..

[14] Xu S., He Q., Tao S., Chen H., Chai Y., Zheng W. (2022). Pig Face Recognition Based on Trapezoid Normalized Pixel Difference Feature and Trimmed Mean Attention Mechanism. IEEE Trans. Instrum. Meas..

[15] Billah M., Wang X., Yu J., Jiang Y. (2022). Real-Time Goat Face Recognition Using Convolutional Neural Network. Comput. Electron. Agric..

[16] Guo Y., Yu Z., Hou Z., Zhang W., Qi G. (2023). Sheep Face Image Dataset and DT-YOLOv5s for Sheep Breed Recognition. Comput. Electron. Agric..

[17] Hansen M.F., Smith M.L., Smith L.N., Salter M.G., Baxter E.M., Farish M., Grieve B. (2018). Towards On-Farm Pig Face Recognition Using Convolutional Neural Networks. Comput. Ind..

[18] Ma C., Deng M., Yin Y. (2024). Pig Face Recognition Based on Improved YOLOv4 Lightweight Neural Network. Inf. Process. Agric..

[19] Wang R., Gao R., Li Q., Dong J. (2023). Pig Face Recognition Based on Metric Learning by Combining a Residual Network and Attention Mechanism. Agriculture.

[20] Wang R., Shi Z., Li Q., Gao R., Zhao C., Feng L. (2021). Pig Face Recognition Model Based on a Cascaded Network. Appl. Eng. Agric..

[21] Guo J., Kong Y., Lin L., Xu L., Feng D., Cao L., Chen J., Ye J., Ye S., Yao Z. (2024). Lightweight Network Based on Fourth Order Runge-Kutta Scheme and Hybrid Attention Module for Pig Face Recognition. Comput. Electron. Agric..

[22] Chen Z., Wu X., Zheng D., Wang Y., Chai J., Zhang T., Wu P., Wei M., Zhou T., Long K. (2025). Single-Nucleus RNA Sequencing Reveals Cellular Transcriptome Features at Different Growth Stages in Porcine Skeletal Muscle. Cells.

[23] Odeh A., Odeh N. (2024). OpenCV and Its Applications in Artificial Intelligent Systems.

[24] Yan B., Fan P., Lei X., Liu Z., Yang F. (2021). A Real-Time Apple Targets Detection Method for Picking Robot Based on Improved YOLOv5. Remote Sens..

[25] Wang T., Hu Y., Yin H. (2026). Enhancing Pig Behavior Recognition in Complex Environments: A Transfer Learning-Assisted YOLO11 Network with Wavelet Convolution and Synergistic Attention. Animals.

[26] Khanam R., Hussain M. (2024). Yolov11: An Overview of the Key Architectural Enhancements. arXiv.

[27] Zhong J., Chen J., Mian A. (2022). DualConv: Dual Convolutional Kernels for Lightweight Deep Neural Networks. IEEE Trans. Neural Netw. Learn. Syst..

[28] Nakano M., Takahashi A., Takahashi S. (2017). Generalized Exponential Moving Average (EMA) Model with Particle Filtering and Anomaly Detection. Expert Syst. Appl..

[29] Tang W., Kang H., Cao Y., Yu P., Han H., Zhang R., Chen K. (2021). M-SEAM-NAM: Multi-Instance Self-Supervised Equivalent Attention Mechanism with Neighborhood Affinity Module for Double Weakly Supervised Segmentation of COVID-19.

[30] Wang H., Guo E., Chen F., Chen P. (2023). Depth Completion in Autonomous Driving: Adaptive Spatial Feature Fusion and Semi-Quantitative Visualization. Appl. Sci..

[31] Chen W., Huang H., Peng S., Zhou C., Zhang C. (2021). YOLO-Face: A Real-Time Face Detector. Vis. Comput..

[32] Liu S., Huang D., Wang Y. (2019). Learning Spatial Fusion for Single-Shot Object Detection. arXiv.

[33] Jocher G., Chaurasia A., Stoken A., Borovec J., Kwon Y., Michael K., Fang J., Wong C., Yifu Z., Montes D. (2022). Ultralytics/Yolov5: V6. 2-Yolov5 Classification Models, Apple M1, Reproducibility, Clearml and Deci.Ai Integrations.

[34] Sohan M., Sai Ram T., Rami Reddy C.V. (2024). A Review on Yolov8 and Its Advancements.

[35] Wang C.-Y., Liao H.-Y.M. (2024). YOLOv1 to YOLOv10: The Fastest and Most Accurate Real-Time Object Detection Systems. arXiv.

[36] Hidayatullah P., Syakrani N., Sholahuddin M.R., Gelar T., Tubagus R. (2025). Yolov8 to Yolo11: A Comprehensive Architecture In-depth Comparative Review. arXiv.

[37] Khanam R., Hussain M. (2025). A Review of YOLOv12: Attention-Based Enhancements vs. Previous Versions. arXiv.

[38] Sun X., Wu P., Hoi S.C. (2018). Face Detection Using Deep Learning: An Improved Faster RCNN Approach. Neurocomputing.

[39] Cao X., Wang H., Wang X., Hu B. (2024). DFS-DETR: Detailed-Feature-Sensitive Detector for Small Object Detection in Aerial Images Using Transformer. Electronics.

[40] Wang C., He W., Nie Y., Guo J., Liu C., Wang Y., Han K. (2023). Gold-YOLO: Efficient Object Detector via Gather-and-Distribute Mechanism. Adv. Neural Inf. Process. Syst..

[41] Zhang H., Li F., Liu S., Zhang L., Su H., Zhu J., Ni L.M., Shum H.-Y. (2022). DINO: DETR with Improved DeNoising Anchor Boxes for End-to-End Object Detection. arXiv.

[42] Selvaraju R.R., Cogswell M., Das A., Vedantam R., Parikh D., Batra D. (2020). Grad-CAM: Visual Explanations from Deep Networks via Gradient-Based Localization. Int. J. Comput. Vis..

